# Comparative Metabolomics Reveals Key Pathways Associated With the Synergistic Killing of Colistin and Sulbactam Combination Against Multidrug-Resistant *Acinetobacter baumannii*

**DOI:** 10.3389/fphar.2019.00754

**Published:** 2019-07-04

**Authors:** Mei-Ling Han, Xiaofen Liu, Tony Velkov, Yu-Wei Lin, Yan Zhu, Darren J. Creek, Christopher K. Barlow, Heidi H. Yu, Zhihui Zhou, Jing Zhang, Jian Li

**Affiliations:** ^1^Biomedicine Discovery Institute, Infection and Immunity Program, Department of Microbiology, Monash University, Clayton, VIC, Australia; ^2^Institute of Antibiotics, Huashan Hospital, Fudan University, Shanghai, China; ^3^Department of Pharmacology & Therapeutics, School of Biomedical Sciences, Faculty of Medicine, Dentistry and Health Sciences, The University of Melbourne, Parkville, VIC, Australia; ^4^Drug Delivery, Disposition and Dynamics, Monash Institute of Pharmaceutical Sciences, Monash University, Parkville, VIC, Australia; ^5^Department of Biochemistry and Molecular Biology, Monash University, Clayton, VIC, Australia; ^6^Department of Infectious Diseases, Sir Run Run Shaw Hospital, Zhejiang University School of Medicine, Hangzhou, China

**Keywords:** polymyxin, β-lactam, combination therapy, lipopolysaccharide, peptidoglycan, metabolomics

## Abstract

**Background:** Polymyxins are a last-line class of antibiotics against multidrug-resistant *Acinetobacter baumannii*. However, polymyxin resistance can emerge with monotherapy, highlighting the need for synergistic combination therapies. Polymyxins in combination with *β*-lactams have shown remarkable synergy against multidrug-resistant *A. baumannii*.

**Methods:** Liquid chromatography–mass spectrometry-based metabolomics was conducted to investigate the metabolic perturbations in an *A. baumannii* clinical isolate, AB090342, in response to colistin (1 mg/L), sulbactam (128 mg/L), and their combination at 1, 4, and 24 h. Metabolomics data were analyzed using univariate and multivariate statistics, and metabolites showing ≥2-fold changes were subjected to pathway analysis.

**Results:** The synergistic activity of colistin–sulbactam combination was initially driven by colistin through perturbation of fatty acid and phospholipid levels at 1 h. Cell wall biosynthesis was perturbed by sulbactam alone and the combination over 24 h; this was demonstrated by the decreased levels of two important precursors, uridine diphosphate-*N*-acetylglucosamine and uridine diphosphate-*N*-acetylmuramate, together with perturbed lysine and amino sugar metabolism. Moreover, sulbactam alone and the combination significantly depleted nucleotide metabolism and the associated arginine biosynthesis, glutamate metabolism, and pentose phosphate pathway. Notably, the colistin–sulbactam combination decreased amino acid and nucleotide levels more dramatically at 4 h compared with both monotherapies.

**Conclusions:** This is the first metabolomics study revealing the time-dependent synergistic activity of colistin and sulbactam against *A. baumannii*, which was largely driven by sulbactam through the inhibition of cell wall biosynthesis. Our mechanistic findings may help optimizing synergistic colistin combinations in patients.

## Introduction

Multidrug-resistant (MDR) *Acinetobacter baumannii* is identified as one of the three “critical priority pathogens” by the World Health Organization that urgently require the development of novel therapies (Dijkshoorn et al., 2007; Fishbain and Peleg, 2010). More worryingly, *A. baumannii* can rapidly develop resistance to all clinically available antibiotics, including the last-line polymyxins (i.e. polymyxin B and colistin) (Mak et al., 2008; Boucher et al., 2009). Polymyxins are a family of cyclic lipopeptides with six L-*α*,*γ*-diaminobutyric acid (Dab) residues, two hydrophobic amino acids (D-Phe and L-Leu), two L-Thr, and an *N*-terminal fatty acyl chain (Velkov et al., 2010). Polymyxins exert their antimicrobial activity through an initial polar interaction with the phosphate groups and a subsequent hydrophobic interaction with the fatty acyl chains of the lipid A component of lipopolysaccharide (LPS) in gram-negative bacterial outer membrane (OM) (Velkov et al., 2010; Rabanal and Cajal, 2017). However, it remains unclear how polymyxins precisely kill bacteria.

Worryingly, polymyxin resistance can rapidly emerge in *A. baumannii*, and novel therapeutic options are urgently needed. The current understanding of polymyxin resistance in *A. baumannii* is largely based on covalent modifications of lipid A phosphate groups with positively charged phosphoethanolamine and/or galactosamine moieties as well as the loss of LPS (Moffatt et al., 2010; Arroyo et al., 2011; Boll et al., 2015). These modifications severely diminish the net negative charge of lipid A, therefore, confer resistance to polymyxins. Synergistic antibiotic combinations are advantageous to reduce the emergence of resistance (Karageorgopoulos and Falagas, 2008; Henry et al., 2015; Nation et al., 2015). Several laboratory studies and clinical case reports have demonstrated that colistin in combination with sulbactam shows synergy against MDR *A. baumannii* (Kempf et al., 2012; Batirel et al., 2014; Leelasupasri et al., 2018). Sulbactam is a *β*-lactamase inhibitor that is typically administered in combination with ampicillin or cefoperazone; however, sulbactam itself has shown efficacy against MDR *A. baumannii* in preclinical and clinical studies (Chu et al., 2013; Jaruratanasirikul et al., 2016; Chen et al., 2017).

The intracellular responses underlying the synergistic effect of colistin–sulbactam combination remain unknown. Metabolomics has been demonstrated as a powerful systems tool for understanding bacterial physiology and mechanisms of antibiotic action and resistance (Henry et al., 2015; Maifiah et al., 2017; Zampieri et al., 2017; Han et al., 2018b). Here, we conducted a metabolomics study to investigate the metabolic perturbations in *A. baumannii* caused by the colistin–sulbactam combination. Our study discovered a time-dependent synergy of colistin–sulbactam combination by perturbing multiple metabolic pathways, in particular the significant inhibition of cell wall biosynthesis.

## Materials and Methods

### Antibiotics, Reagents, and Bacterial Isolate

Colistin (sulfate; CAS# 1264-72-8) and sulbactam (CAS# 68373-14-8) were obtained from Sigma-Aldrich (Saint Louis, USA). Stock solutions (5.12 mg/ml of each) were prepared using Milli-Q water and filtered through 0.22-μm syringe filters (Sartorius, Melbourne, VIC, Australia). All organic solvents used in metabolite sample extraction and the mobile phase of liquid chromatography (LC) were LC–mass spectrometry (LC-MS) grade and purchased from Merck Millipore (Bayswater, VIC, Australia).

*A. baumannii* AB090342 was collected from a patient (60–65 years old) with ventilator-associated pneumonia who received intravenous colistimethate sodium (150-mg colistin base activity every 12 h for 10 days) (Han et al., 2018a). The patient was enrolled in a clinical study that was approved by the institutional review board of Sir Run Run Shaw Hospital (Zhejiang, China) and Huashan Hospital (Shanghai, China), and an informed consent form was obtained from the patient before the study. Minimum inhibitory concentrations of colistin (0.5 mg/L) and sulbactam (128 mg/L) were determined by broth microdilution.

### Bacterial Culture for the Metabolomics Experiment

Culture of *A. baumannii* AB090342 was prepared from frozen stock (−80°C) on Mueller–Hinton agar plates and incubated at 37°C for approximately 18 h. A single colony was then inoculated into 10 ml of cation-adjusted Mueller–Hinton broth (CaMHB) with an incubation at 37°C for 18 h in a shaking water bath (180 rpm). Overnight culture (5 ml) was then diluted by 1:100 with 495-ml CaMHB and grown to an optical density at 600 nm (OD_600_) of 0.50 ± 0.02 to achieve an early logarithmic growth phase (i.e. ∼10^8^ cfu/ml). For each sample, 100 ml of the early logarithmic bacterial culture was treated with colistin (1 mg/L), sulbactam (128 mg/L), or the combination (colistin at 1 mg/L and sulbactam at 128 mg/L) for 1, 4, and 24 h, and the untreated culture was used as the control. Concentrations of colistin (1 mg/L) and sulbactam (128 mg/L) were chosen based on an *in vitro* static time-kill study to ensure both synergistic activity and sufficient bacterial cell numbers for the metabolomics study (**Figure S1**). For each condition, five biological replicates were prepared independently on different days from different colonies on agar plates.

### Metabolite Sample Preparation

Cellular metabolites were extracted from *A. baumannii* AB090342 as previously described (Han et al., 2018b). Briefly, bacterial culture samples (20 ml) from both treated and untreated groups were collected at 0, 1, 4, and 24 h and were transferred immediately into a dry ice–ethanol bath for quenching to stop metabolic processes. The culture was then normalized according to OD_600_ at 0.50 ± 0.02 using fresh CaMHB broth. Bacterial cell pellets were obtained by centrifuging 15 ml of the normalized culture at 3,220 × *g* and 4°C for 10 min. Cell pellets were then washed twice with 2-ml 0.9% saline and resuspended in 0.5-ml extraction solvent (CHCl_3_/MeOH/H_2_O, 1:3:1, v/v) containing 1-μM generic internal standards (CHAPS, CAPS, PIPES, and TRIS). Three freeze–thaw cycles were performed in liquid nitrogen to ensure bacterial lysis and metabolite extraction efficiency. After centrifugation at 3,220 × *g* and 4°C for 10 min, 300-μl supernatant containing extracted metabolites was collected and further centrifuged at 14,000 × *g* for 10 min to obtain 200-μl particle-free supernatant for LC-MS analysis.

### Metabolite Profiling Using Liquid Chromatography–Mass Spectrometry

Metabolite samples were analyzed using a Q-Exactive Orbitrap mass spectrometer (Thermo Fisher), coupled to a Dionex high-performance liquid chromatography (U3000 HPLC, Thermo Fisher) (Han et al., 2018b). In brief, the MS system was operated at 35,000 resolution in both positive and negative electro-spray ionization mode with a detection range of 85–1,275 *m/z*. Metabolite samples were eluted through a ZIC-pHILIC column by a multiple-gradient step containing acetonitrile in mobile phase A and 20-mM ammonium carbonate in mobile phase B. The gradient started from 80% mobile phase A and proceeded as a linear gradient to 50% over 15 min at 0.3 ml/min. Over the next 3 min, mobile phase A was changed to 5%, followed by a washing step with 95% mobile phase B and 5% mobile phase A for 3 min and re-equilibration with 80% mobile phase A and 20% mobile phase B for 8 min. The injection volume was 10 μl, and all of the samples were analyzed within the same LC-MS batch. A pooled biological quality control sample consisted of 10 μl of each sample was analyzed periodically throughout the batch to assess the chromatographic peaks, signal reproducibility, and analyte stability. Eight standard samples containing the mixture of more than 300 authentic standards were also analyzed within the batch to assist metabolite identification.

### Metabolomics Data Processing, Statistical Analysis, and Pathway Mapping

Metabolomics raw data were initially converted to mzXML and followed by feature detection with XCMS and annotated using mzMatch (Smith et al., 2006; Scheltema et al., 2011). The mzMatch data were then filtered, identified, quantified, and visualized in IDEOM using default settings (http://mzmatch.sourceforge.net/ideom.php) (Creek et al., 2012). Relative intensities (peak height) of features were normalized according to the median height of all putatively identified peaks and log transformed before statistical analysis. Principal component analysis (PCA), log-transformed relative intensities, and median relative standard deviation of both quality control and sample groups (<30%) were analyzed to assess the precision of metabolomics data (**Figure S2**). Global metabolic variations due to antibiotic treatments at each time point were visualized using PCA score plots. Univariate statistics was conducted using one-way analysis of variance (ANOVA) for multiple group comparisons, and the *p*-value was corrected by Benjamini–Hochberg method to keep the false discovery rates (FDR) < 0.05 (Benjamini and Hochberg, 1995). Metabolites were identified by accurate mass and retention time with authentic standards as indicated by IDEOM confidence score of 9 or 10 (corresponding to metabolomics standards initiative level 1 based on Metabolomics Standards Initiative Guidelines), or by accurate mass and predicted retention time to achieve an IDEOM confidence score of 6 or greater (metabolomics standards initiative level 2/3) (Salek et al., 2013). Metabolites that showed ≥2-fold change were further examined and subjected to pathway analysis according to the Kyoto Encyclopedia of Genes and Genomes (KEGG) pathway and Ecocyc (Kanehisa and Goto, 2000; Keseler et al., 2005).

## Results

### Global Metabolic Perturbations due to Treatments with Colistin and Sulbactam Monotherapy and the Combination

**Figure 1:**
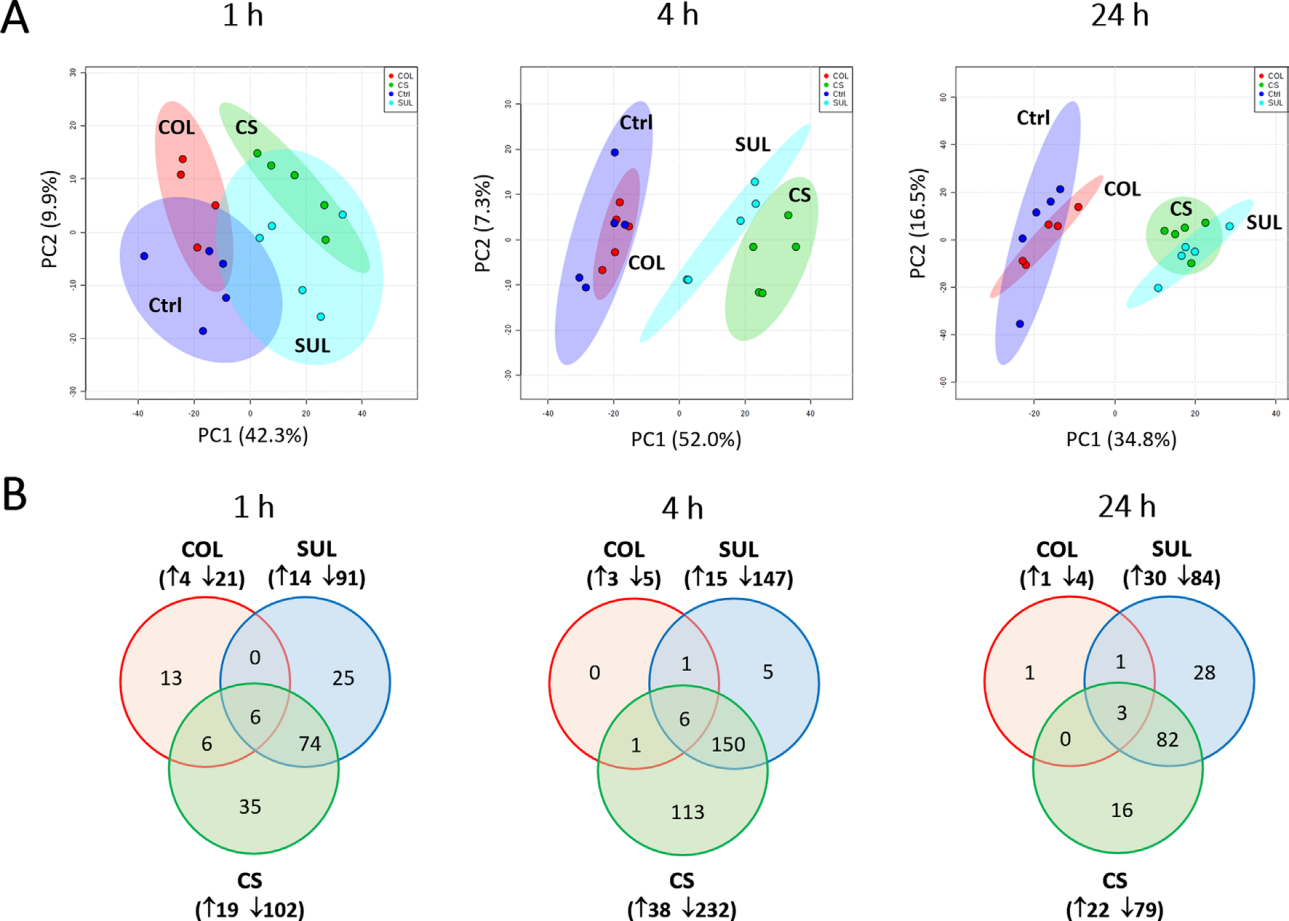
Multivariate and univariate analyses of global metabolic changes. **(A)** Principal component analysis (PCA) score plots of the first two principal components for metabolite levels in *A. baumannii* AB090342 after treatments with colistin alone, sulbactam alone, and the combination at 1, 4, and 24 h. Each dataset represents a total of 20 samples with five biological replicates under each condition. Blue = untreated control (Ctrl); red = colistin alone (COL); cyan = sulbactam alone (SUL); green = the combination (CS). **(B)** Venn diagrams show the number of significantly affected metabolites by each treatment at 1, 4, and 24 h. Significant metabolites were selected based on false discovery rates (FDR) < 0.05 and |log_2_FC| ≥ 1 (one-way ANOVA). The up and down arrows indicate metabolites that were significantly increased and decreased, respectively.

Our untargeted metabolomics analyses resulted in 1,015, 1,035, and 1,036 putatively identified metabolites in *A. baumannii* AB090342 at 1, 4, and 24 h, respectively, involving in multiple biochemical pathways, such as amino acid, carbohydrate, lipid, and nucleotide metabolism (**Figure S3**, **Supplementary dataset S1**). The PCA score plots showed that the colistin–sulbactam combination induced the most significant metabolic changes at 4 h (PC1 = 52.0%) compared with 1 h (PC1 = 42.3%) and 24 h (PC1 = 34.8%) (**Figure 1A**). In general, sulbactam monotherapy and the combination led to more metabolic variations over 24 h than colistin alone that was close to the untreated control. From Venn diagrams, the combination resulted in 121 (11.9%), 270 (26.1%), and 101 (9.7%) significant metabolites [false discovery rates (FDR) < 0.05, fold change (FC) ≥2] at 1, 4, and 24 h, respectively. Similarly, sulbactam monotherapy led to 105 (10.3%), 162 (15.7%), and 114 (11.0%) significantly affected metabolites across all three time points accordingly. However, colistin alone caused minimal metabolic perturbations over 24 h, with only 25 (2.5%), 8 (0.8%), and 5 (0.5%) significant metabolites at 1, 4, and 24 h, respectively (**Figure 1B**). Sulbactam monotherapy and the combination treatment shared a number of common metabolic perturbations over 24 h, suggesting that the synergistic killing of the colistin–sulbactam combination was largely driven by sulbactam. Notably, the colistin–sulbactam combination treatment showed time-dependent synergy by uniquely affecting 113 significantly affected metabolites at 4 h, while only 35 and 16 at 1 and 24 h, respectively (**Figure 1B**).

**Figure 2:**
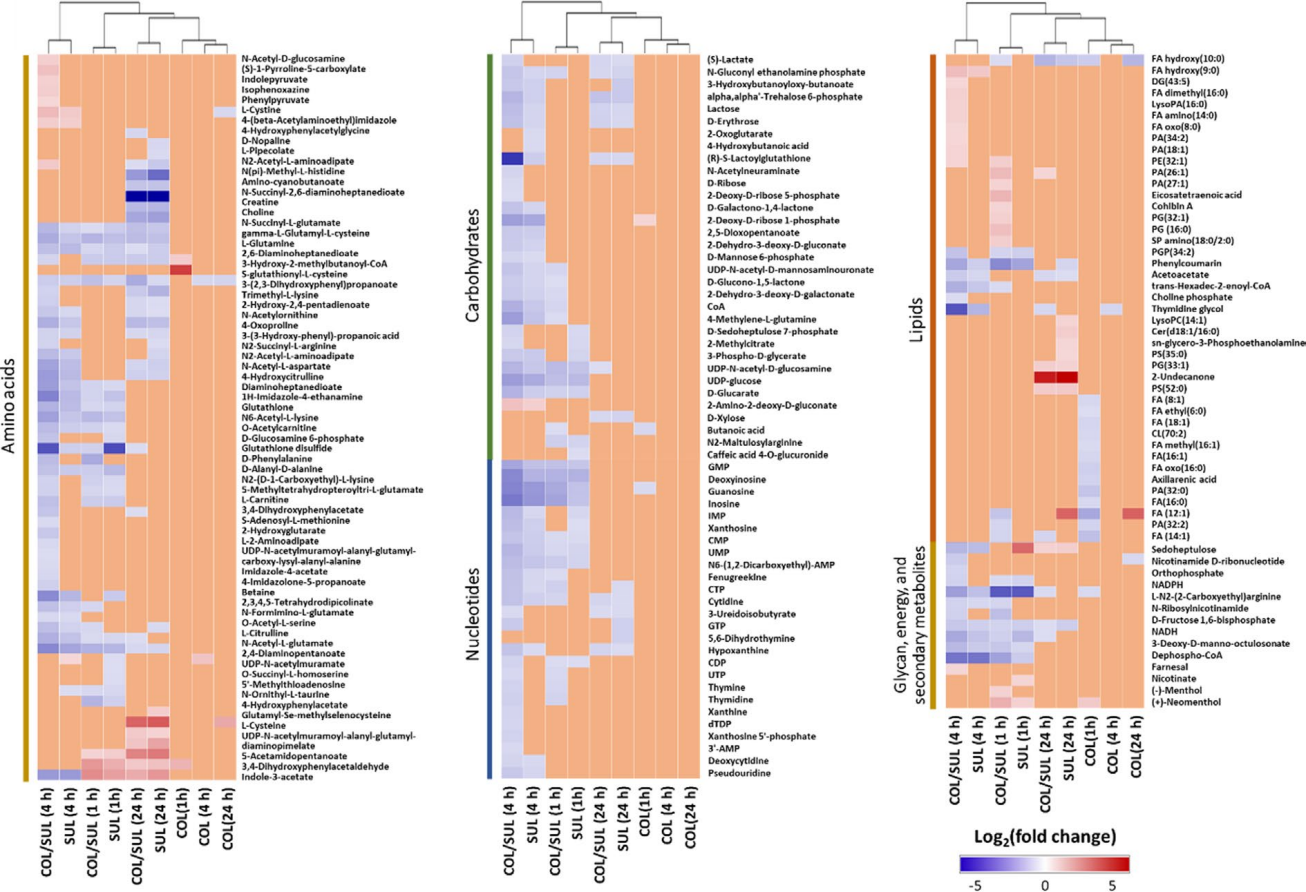
Clustered heat map profiles of the relative abundance for significantly affected metabolites in *A. baumannii* AB090342. Metabolites are grouped into different classes: amino acids, carbohydrates, lipids, nucleotides, glycan, energy, and secondary metabolites. The colors indicate the relative abundance of significantly affected metabolites by different treatments [colistin alone (COL), sulbactam alone (SUL), and the combination (COL/SUL)] at 1, 4, and 24 h, compared with the untreated control samples. Blue = significant decrease (log_2_FC ≤ −1), red = significant increase (log_2_FC ≥ 1), and orange = not significant.

**Figure 3:**
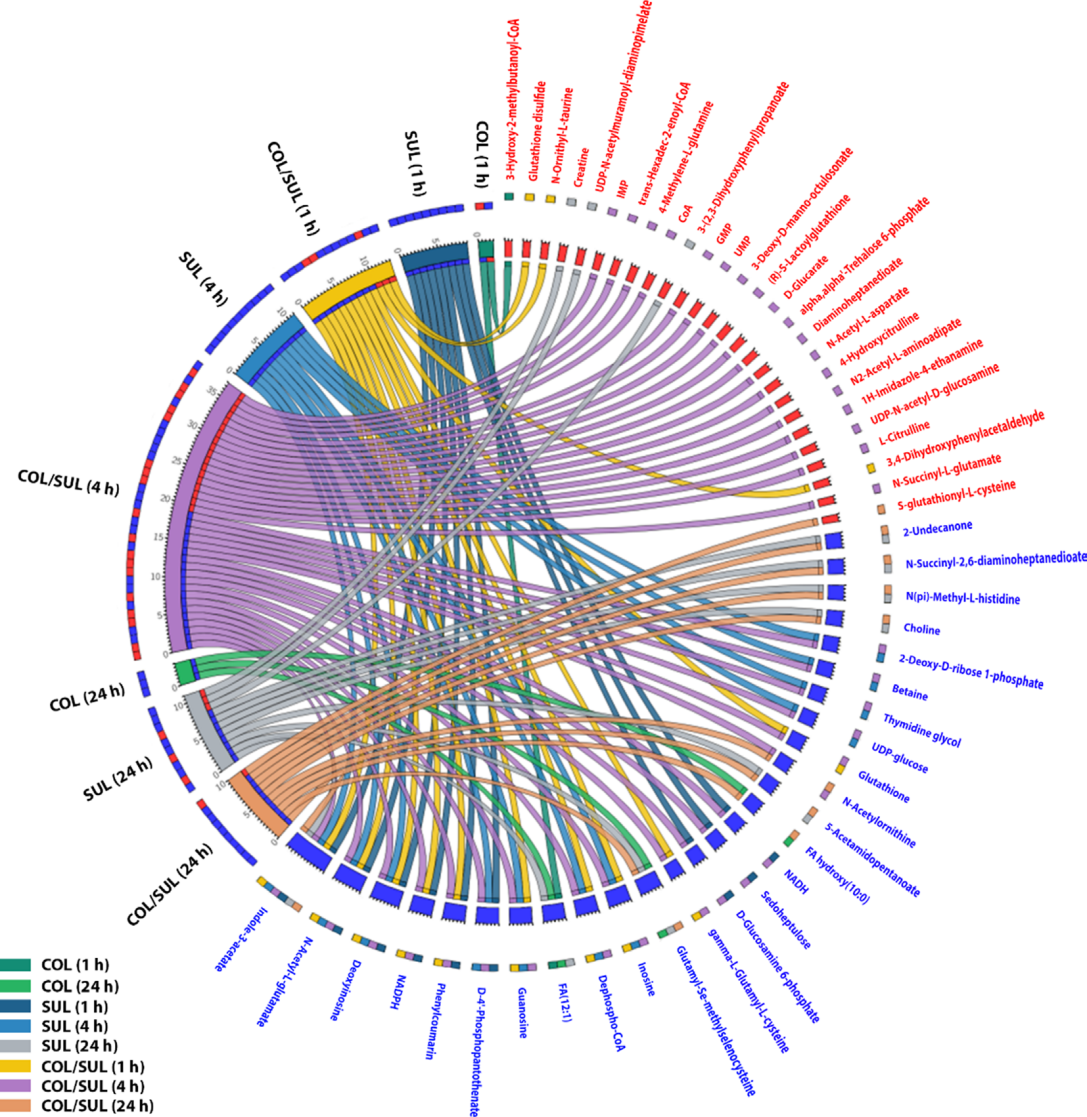
Significantly affected metabolites in *A. baumannii* AB090342 after the treatments with colistin (COL), sulbactam (SUL), and the combination (COL/SUL) over 24 h. Metabolites with |log_2_FC| ≥ 2 were chosen for correlation analysis. The bipartite graph shows the correlations of all three treatments at 1, 4, and 24 h (left) and significantly affected metabolites (right). Metabolite names and labels in red indicate unique metabolic changes due to a specific treatment, while those in blue show common metabolic perturbations between at least two conditions.

**Figure 4:**
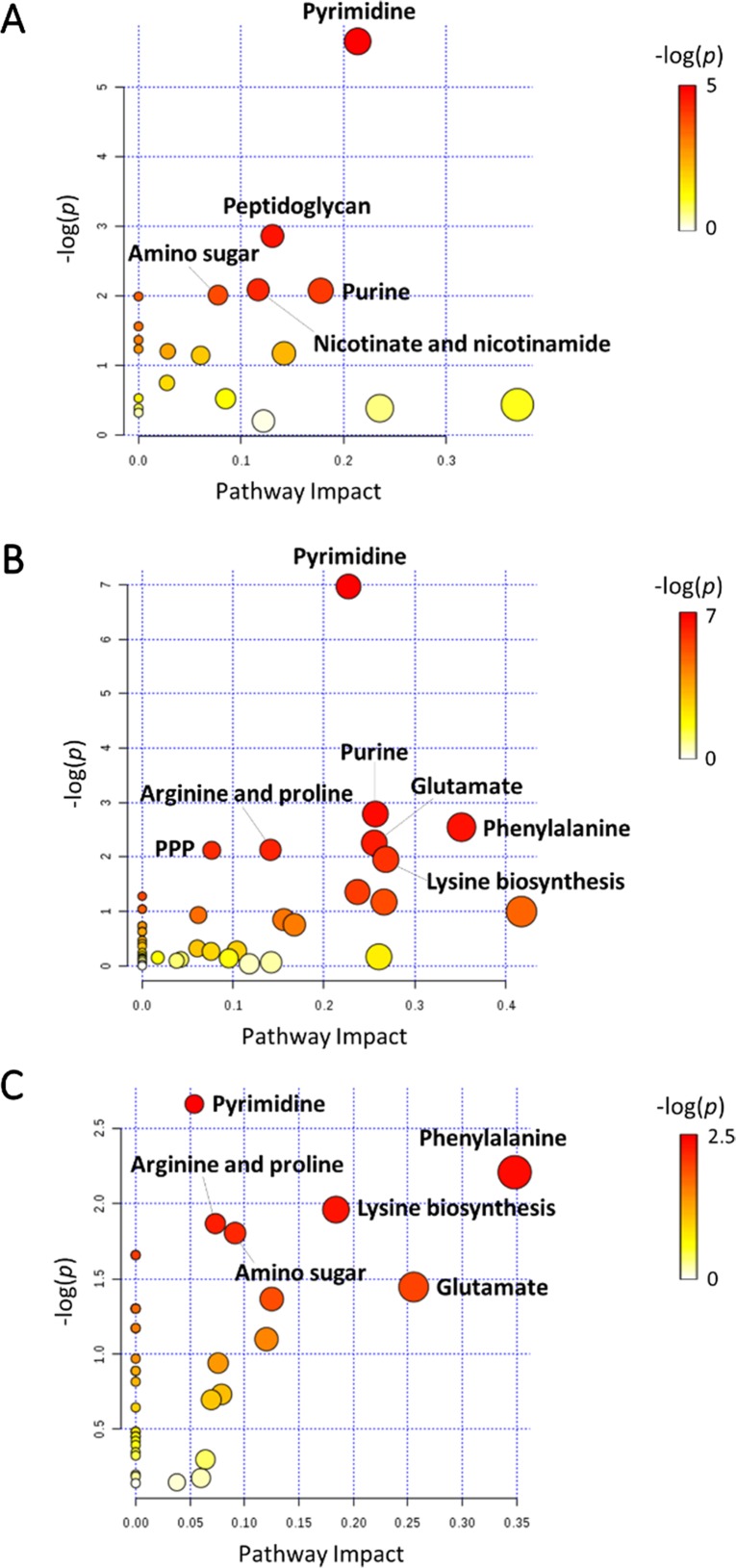
Pathway analysis of significantly affected metabolites in *A. baumannii* AB090342 following the treatments with colistin, sulbactam alone, and the combination at **(A)** 1 h, **(B)** 4 h, and **(C)** 24 h (FDR < 0.05 and |log_2_FC| ≥ 1, one-way ANOVA). The pathway enrichment analysis was based on KEGG Pathway (http://www.genome.jp/kegg/pathway.html) with reference to *Escherichia coli* K-12. PPP, pentose phosphate pathway.

In general, the heat map showed that sulbactam alone and the combination dramatically decreased amino acid, carbohydrate, nucleotide, and peptide metabolism over 24 h, especially at 4 h (**Figures 2**, **S4**, **S5**, and **Supplementary dataset S2**). In particular, the bipartite graph highlights a few key metabolites [e.g. uridine diphosphate (UDP)-glucose, 2-deoxy-D-ribose 1-phosphate, D-glucosamine 6-phosphate, sedoheptulose, dephospho-CoA, D-phosphopantothenate, NADH, NADPH, inosine, deoxyinosine, thymidine glycol, guanosine, *N*-acetyl-L-glutamate, and indoleacetate] that were commonly perturbed by both sulbactam alone and the combination at 4 h (**Figure 3**). Interestingly, a number of metabolites (e.g. UDP-*N*-acetyl-D-glucosamine, CoA, D-glucarate, *α,α‘*-trehalose 6-phosphate, IMP, GMP, UMP, L-citrulline, 4-hydroxycitrulline, *N*-succinyl-L-glutamate, *N*-acetyl-L-aspartate) were more significantly decreased in their abundance by the combination compared with each monotherapy at 4 h (**Figures 2** and **3**). Moreover, the pathway enrichment analysis indicated that pyrimidine, purine, peptidoglycan, amino sugar, arginine, lysine, glutamate, and phenylalanine metabolism were significantly depleted by the combination (**Figures 4** and **S6**). Whereas the lipid levels were increased by the combination mainly at 1 and 4 h, sulbactam alone only affected lipid metabolism at 24 h. Although colistin alone resulted in minimal metabolic perturbations across all three time points, the levels of lipids were significantly decreased at 1 h (**Figures 2** and **S4**).

### Combination Therapy Primarily Perturbed Peptidoglycan Biosynthesis

**Figure 5:**
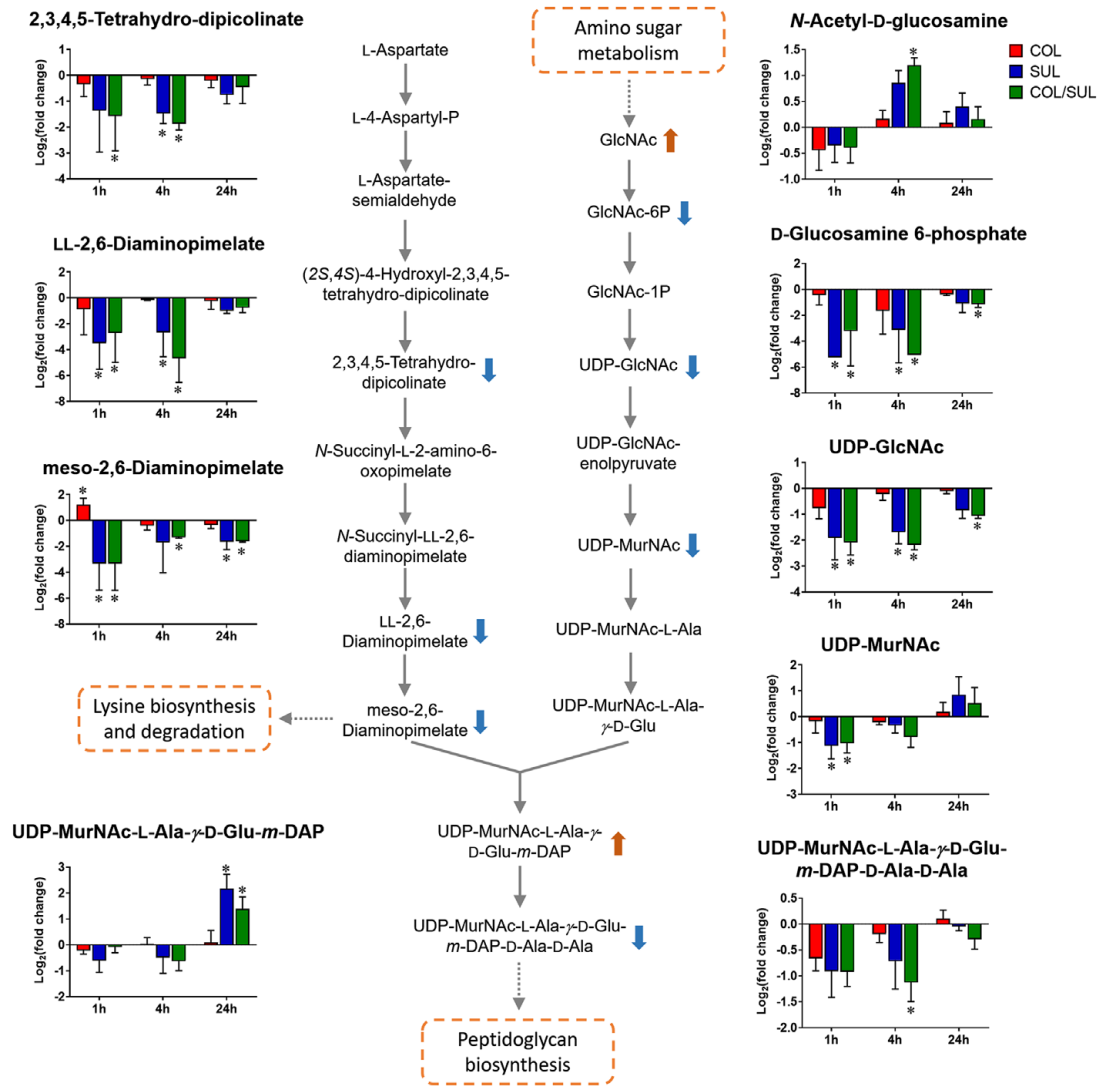
Metabolic changes in peptidoglycan biosynthesis and the related lysine biosynthesis in *A. baumannii* AB090342 after the treatments with colistin (COL, red), sulbactam (SUL, blue), and the combination (COL/SUL, green) compared with the untreated control at 1, 4, and 24 h. The pathway flow charts were adapted from KEGG Pathway (http://www.genome.jp/kegg/pathway.html) with reference to *E. coli* K-12. The bar charts show log_2_FC of metabolic changes compared with the control. Orange and blue arrows indicate the metabolites that were significantly increased and decreased, respectively. *FDR < 0.05 and |log_2_FC| ≥ 1 (one-way ANOVA).

Sulbactam alone and the combination resulted in significant metabolic variations in the pathways associated with peptidoglycan biosynthesis over 24 h (**Figure 5**). More specifically, a key peptidoglycan biosynthesis cytoplasmic precursor, UDP-MurNAc-L-Ala-*γ*-D-Glu-*m*-DAP-D-Ala-D-Ala, was significantly decreased in abundance (log_2_FC < −1) at 1 and 4 h. In line with this event, we observed an accumulation in its upstream metabolite, UDP-MurNAc-L-Ala-*γ*-D-Glu-*m*-DAP, at 24 h (**Figures 2** and **5**). UDP-GlcNAc and UDP-MurNAc are two important metabolites in amino sugar metabolism that serve as precursors in peptidoglycan synthesis (Vollmer et al., 2008; Das et al., 2011). Our metabolomics data showed a dramatic decrease in the abundance of UDP-GlcNAc in response to sulbactam alone (log_2_FC = −1.7, −1.6, and −0.8) and the combination (log_2_FC = −2.0, −2.1, and −1.0) at 1, 4, and 24 h, respectively. The level of UDP-MurNAc at 1 h was also decreased following both sulbactam alone and the combination (log_2_FC = −1.0 and −0.9, respectively). In addition, another two metabolites in amino sugar metabolism, GlcNAc (log_2_FC > 1) and GlcNAc-6P (log_2_FC < −1), were also dramatically affected following both sulbactam and the combination. Lysine biosynthesis is another pathway associated with peptidoglycan biosynthesis (Vollmer et al., 2008); consistently, our data revealed significant depletion of 2,3,4,5-tetrahydro-dipicolinate, L,L-2,6-diaminopimelate, and *meso*-2,6-diaminopimelate due to the treatments of sulbactam alone and the combination over 24 h (log_2_FC < −1) (**Figure 5**).

### Combination Therapy Decreased Nucleotide and Amino Acid Metabolism

**Figure 6:**
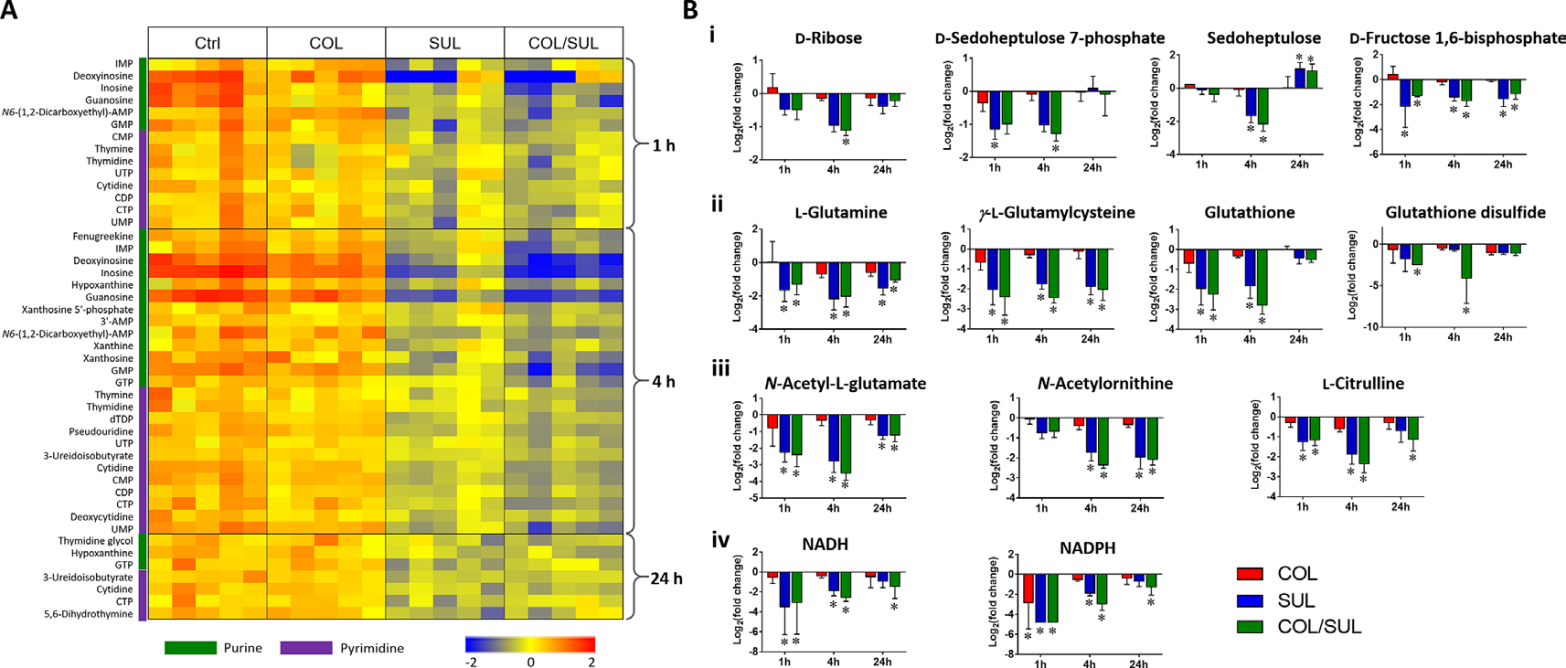
**(A)** Heat map profiles of the relative abundance of nucleotide metabolites and **(B)** bar charts showed metabolic changes in (i) pentose phosphate pathway, (ii) glutamate metabolism, (iii) arginine biosynthesis, and (iv) energy metabolism that are associated with nucleotide metabolism under the treatments of colistin (COL, red), sulbactam (SUL, blue), and the combination (COL/SUL, green) at 1, 4, and 24 h in *A. baumannii* AB090342. *FDR < 0.05 and |log_2_FC| ≥ 1 (one-way ANOVA).

Metabolites related to purine (e.g. IMP, AMP, GMP, inosine, hypoxanthine, xanthosine, xanthine, and guanosine) and pyrimidine (e.g. UMP, UTP, CDP, CMP, cytidine, cytosine, dTDP, dTMP, thymidine, and thymine) metabolism were also significantly decreased (log_2_FC < −1) in their abundance due to sulbactam alone or the combination over 24 h, especially at 4 h (**Figures 2**, **6A**, and **S7**). Notably, the combination induced more dramatic changes in nucleotide metabolism than sulbactam alone at 4 h (**Figures 3** and **6A**); in particular, the combination exclusively perturbed the levels of IMP, GMP, and UMP at 4 h (**Figure 2**). At all three time points, sulbactam alone and the combination significantly depleted metabolites in glutamate metabolism (i.e. L-glutamine, L-*γ*-glutamylcysteine), arginine biosynthesis (i.e. *N*-acetyl-L-glutamate, *N*-acetyl-L-ornithine, and L-citrulline), and pentose phosphate pathway (PPP) (i.e. D-ribose, D-sedoheptulose 7-phosphate, sedoheptulose, and D-fructose 1,6-biphosphate), which are related to nucleotide metabolism (**Figures 6B i–iii** and **S8**). Furthermore, our results showed significantly decreased abundance of three major metabolites associated with glutamate metabolism, L-γ-glutamylcysteine, glutathione, and glutathione disulphide (GSSG), following sulbactam alone or the combination at 1 and 4 h (**Figures 6Bii** and **S8** **)**. Interestingly, the relative abundance of two nucleotide-derived metabolites related to redox status, NADH and NADPH, were dramatically decreased by the combination as early as 1 h and lasted until 24 h. Notably, sulbactam alone also decreased the levels of NADH and NADPH at 1 and 4 h, while colistin alone only decreased NADPH level at 1 h (**Figure 6Biv**).

### Combination Therapy Perturbed Lipid Levels

**Figure 7:**
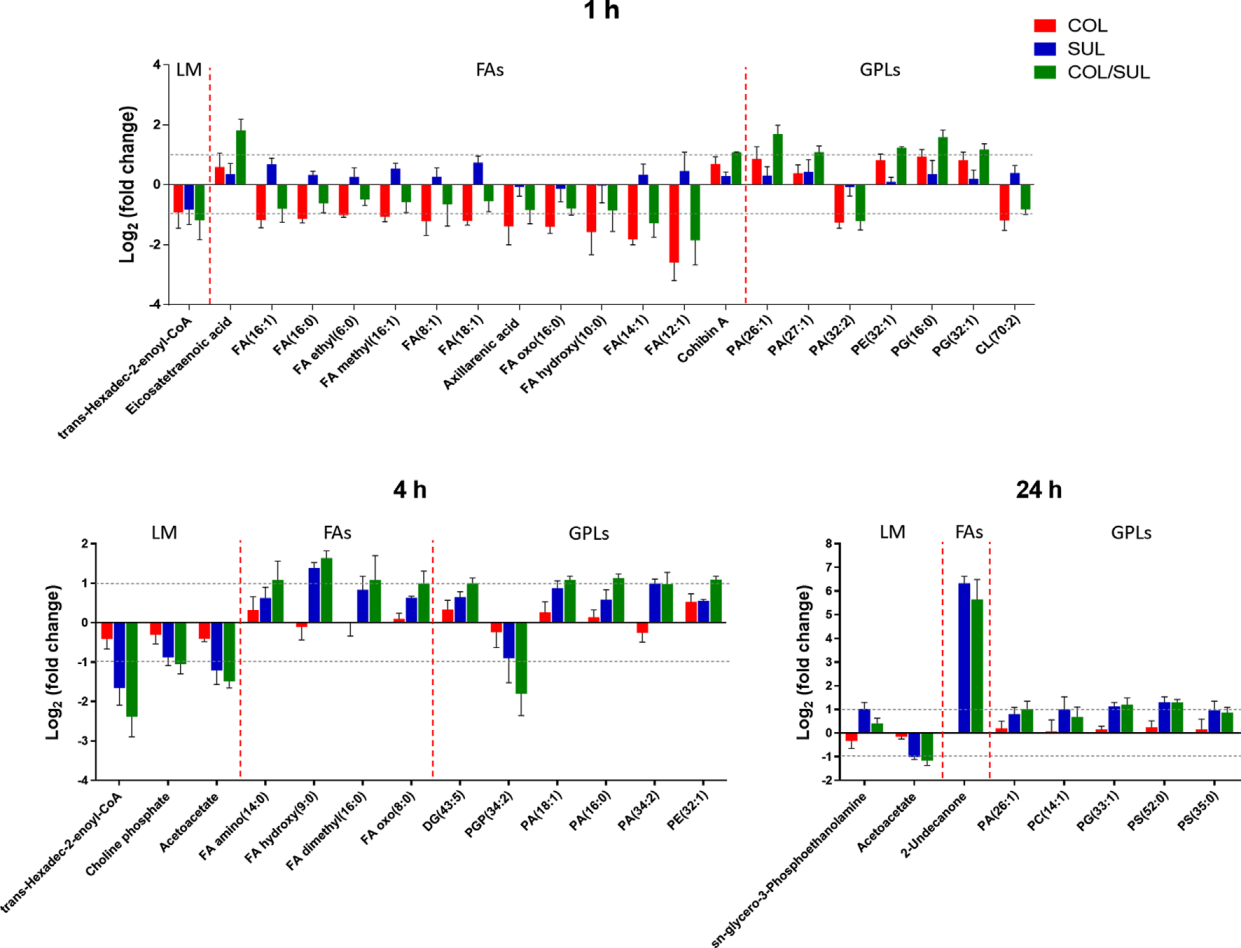
Major lipid classes significantly perturbed following the treatment with colistin alone (COL, red), sulbactam alone (SUL, blue), and the combination (COL/SUL, green) at 1, 4, and 24 h. Lipids were putatively identified based on the accurate mass. LM, lipid metabolism; FAs, fatty acids; GPLs, glycerophospholipids.

At 1 h, colistin alone induced a dramatic decrease in the abundance of fatty acid levels, while the combination caused a similar but less dramatic decrease in fatty acid levels (**Figure 7**). At 1 h, both colistin alone and the combination significantly enriched a number of phospholipids [e.g. phosphatidic acid (PA)(26:1), PA(27:1), PE(32:1), PG(16:0), and PG(32:1)] but decreased the levels of PA(32:2) and CL(70:2). In contrast, at 4 and 24 h, colistin alone induced minimal changes in lipid levels; however, sulbactam alone and the combination increased both fatty acid and phospholipid levels (**Figure 7**). In particular, sulbactam alone and the combination increased the total phosphatidylglycerol (PG) level at all three time points, especially at 24 h (FC = 1.8, *p* < 0.01), while the total phosphatidylethanolamine (PE) level increased only at 1 and 4 h. However, the level of total cardiolipin (CL) was not significantly affected by either sulbactam alone or the combination therapy over 24 h (**Figure S9**).

## Discussion

Preclinical and clinical studies have demonstrated synergistic killing by the combination of colistin with sulbactam against MDR *A. baumannii* (Kempf et al., 2012; Batirel et al., 2014; Leelasupasri et al., 2018). To the best of our knowledge, the present study is the first to investigate metabolic responses to colistin, sulbactam, and the combination using a systems approach. A clinical *A. baumannii* isolate AB090342 obtained from a patient with ventilator-associated pneumonia was examined (Han et al., 2018a). Our metabolomics data discovered 1) the sulbactam-driven synergistic killing by the combination; 2) time-dependent metabolic perturbations; 3) synergistic inhibition of cell wall biosynthesis and the associated lysine biosynthesis and amino sugar pathways; and 4) significant depletion in nucleotide metabolism, PPP, and the associated glutamate and arginine biosynthetic pathways.

Colistin exerts its antimicrobial activity through an initial interaction with LPS on the gram-negative membrane, resulting in the OM disorganization and phospholipid exchange (Velkov et al., 2013; Rabanal and Cajal, 2017). Following colistin treatment, the significantly decreased fatty acid and increased phospholipid levels observed in *A. baumannii* AB090342 (**Figure 7**) suggest that the synergistic killing of the colistin–sulbactam combination was associated with perturbations of the OM lipid composition caused by colistin (**Figures 4**, **7**, and **S9**). Lack of perturbations on lipid metabolism by colistin at 4 and 24 h indicated that the OM remodelling due to colistin treatment was rapid. Our results are consistent with the literature that colistin significantly perturbed OM asymmetry and upregulated the expression of Mla and Lol systems that are responsible for membrane biogenesis in *A. baumannii* (Henry et al., 2015; Maifiah et al., 2017). In contrast, sulbactam monotherapy did not affect lipid levels at 1 h but dramatically decreased metabolite levels in nucleotide, carbohydrate, amino acid, and peptide metabolism. It is believed that the mechanism of sulbactam antibacterial activity involves the inhibition of cell wall biosynthesis through binding to penicillin-binding proteins 1 and 3 (Urban et al., 1995; Papp-Wallace et al., 2012; Cho et al., 2014; Penwell et al., 2015). In line with this, our metabolomics data showed that sulbactam alone caused considerable decrease in metabolite levels associated with peptidoglycan biosynthesis over 24 h (**Figure 5**). Clearly, cell envelope biogenesis is targeted by both colistin and sulbactam synergistically in a time-dependent manner.

Metabolic perturbations caused by sulbactam alone and the combination are dramatic and largely common over 24 h, indicating that the synergistic action by the combination was mainly driven by sulbactam. Sulbactam alone and the combination dramatically depleted pyrimidine and purine metabolism, suggesting a diminishing pool of nucleotide building blocks (**Figures 5** and **S7**). Furthermore, the marked decreases in arginine biosynthesis pathway and glutamate metabolism compounded the impaired nucleotide metabolism (Belenky et al., 2015). Remarkably, compared with colistin and sulbactam alone, pyrimidine and purine metabolism and the associated lysine, glutamate, and PPP pathways were more severely depleted at 4 h by the combination, indicative of a time-dependent synergistic killing by the combination therapy (**Figures 2**, **3**, and **5**). In fact, the combination therapy showed synergistic killing as early as 1 h and lasted for at least 24 h, as demonstrated by the significantly perturbed lipid levels, decreased nucleotide and amino acid metabolism over 24 h (**Figures 2**, **4**, and **S5**). Particularly, peptidoglycan biosynthesis and the related arginine and amino sugar metabolism were significantly perturbed by the combination and sulbactam alone over 24 h (**Figure 6**), which is in line with the current literature on the mechanism of sulbactam bacterial killing (Papp-Wallace et al., 2012; Penwell et al., 2015).

Lipid A modification pathways were not inhibited by the combination of colistin and sulbactam or the latter alone, demonstrating that the synergistic killing was not due to the inhibition of colistin resistance. This finding was consistent with a recent metabolomics study for the combination of polymyxin B and doripenem against *A. baumannii* (Maifiah et al., 2017). Collectively, the combination synergy between polymyxins and *β*-lactams is mainly through the inhibition of nucleotide metabolism and cell wall biosynthesis (Cho et al., 2014). Furthermore, it has been reported that most antibiotics kill bacteria *via* the production of free radicals and oxidative stress (Belenky et al., 2015). The decreased glutathione and GSSG pools by each antibiotic and the combination in the present study (**Figure 6Bii**) are indicative of oxidative stress (Masip et al., 2006; Dwyer et al., 2014); moreover, the perturbed metabolite levels in PPP and NAD metabolites also indicated an imbalanced redox status within bacterial cells (Ying, 2008).

## Conclusion

Taken together, our metabolomics study demonstrated a time-dependent synergistic activity of colistin–sulbactam combination against *A. baumannii*, which was initially driven by colistin but predominantly by sulbactam afterward. Colistin-induced OM remodelling was rapid, and cell envelope biogenesis was a major target by the synergistic combination of colistin and sulbactam. These mechanistic findings have significant potential in optimizing the combination therapy of colistin and sulbactam in patients using pharmacokinetics/pharmacodynamics.

## Data Availability Statement

All the raw data will be provided upon request. Requests to access the datasets should be directed to meiling.han@monash.edu.

## Ethics Statement

The patient was enrolled in a clinical study which was approved by the institutional review board of Sir Run Run Shaw Hospital (Zhejiang, China) and Huashan Hospital (Shanghai, China), and an Informed Consent Form was obtained from the patient before the study.

## Author Contributions

JZ and JL conceived the project, and all authors were involved in the design of the experiments. M-LH and XL performed the experiments, and M-LH, XL, TV, Y-WL, YZ, DC and CB analyzed the results. All authors reviewed the manuscript.

## Funding

This research was supported by a research grant from the National Natural Science Foundation of China (NSFC 81628015). M-LH and Y-WL are recipients of the 2018 Faculty Bridging Fellowship, Monash University. JL is an Australian National Health and Medical Research Council Principal Research Fellow. TV and DC are Australian National Health and Medical Research Council Career Development Research Fellows.

## Conflict of Interest Statement

The authors declare that the research was conducted in the absence of any commercial or financial relationships that could be construed as a potential conflict of interest.
