# Supplementary material for: Comparative Metabolomics Reveals Key Pathways Associated With the Synergistic Killing of Colistin and Sulbactam Combination Against Multidrug-Resistant Acinetobacter baumannii
Source: Front Pharmacol. 2019 Jul 4;10:754. doi: 10.3389/fphar.2019.00754 (PMC6620532; doi:10.3389/fphar.2019.00754)
Supplement: Supplementary file 3 [file Presentation_1.pdf]

Supporting Information

Comparative metabolomics reveals key pathways associated with the synergistic killing of colistin and sulbactam combination against multidrug-resistant *Acinetobacter baumannii*

Mei-Ling Han[1,2,#], Xiaofen Liu[2,#], Tony Velkov[3], Yu-Wei Lin[1], Yan Zhu[1], Darren J. Creek[4], Christopher K. Barlow[5], Heidi H. Yu[1], Zhihui Zhou[6], Jing Zhang[2,*], Jian Li[1,2,*]

[1]Biomedicine Discovery Institute, Infection and Immunity Program, Department of Microbiology, Monash University, Clayton, Victoria 3800, Australia.

[2]Institute of Antibiotics, Huashan Hospital, Fudan University, Shanghai 200040, China.

[3]Department of Pharmacology & Therapeutics, School of Biomedical Sciences, Faculty of Medicine, Dentistry and Health Sciences, The University of Melbourne, Parkville, Victoria 3010, Australia

[4]Drug Delivery, Disposition and Dynamics, Monash Institute of Pharmaceutical Sciences, Monash University, 381 Royal Parade, Parkville, Victoria 3052, Australia.

[5]Department of Biochemistry and Molecular Biology, Monash University, Clayton, Victoria 3800, Australia.

[6]Department of Infectious Diseases, Sir Run Run Shaw Hospital, Zhejiang University School of Medicine, Hangzhou, China.

[#]Contributed equally to this work.

*To whom correspondence should be addressed to: jian.li@monash.edu or zhangj_fudan@aliyun.com

Running title: Colistin and sulbactam against *Acinetobacter baumannii*

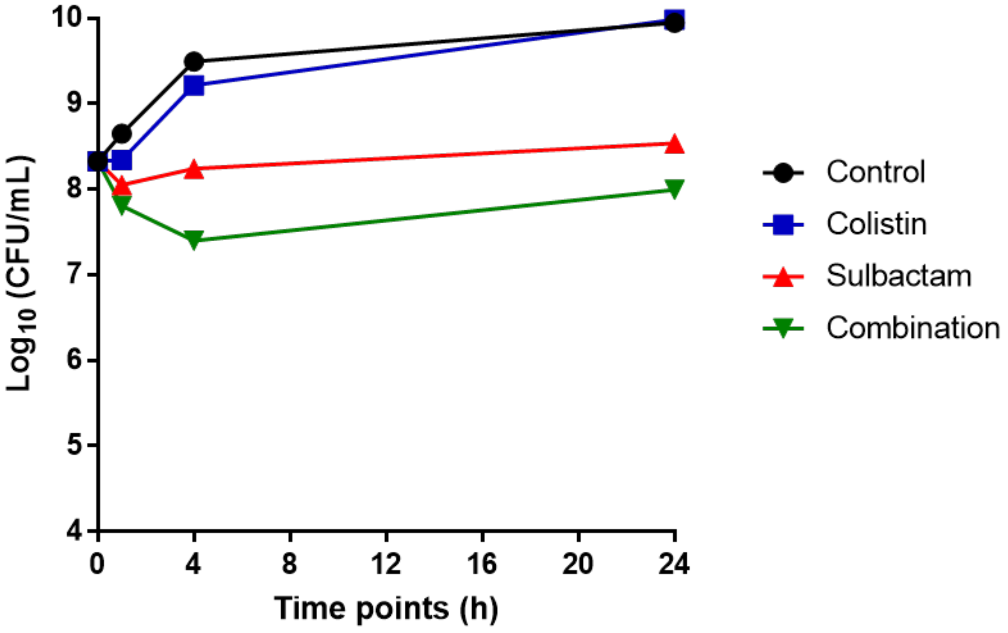

Figure S1. Time-kill kinetics of colistin alone, sulbactam alone and the combination against *A. baumannii* 090342 at 1, 4, and 24 h. The concentrations of colistin and sulbactam were 1 mg/L (2 × MIC) and 128 mg/L (1 × MIC), respectively.

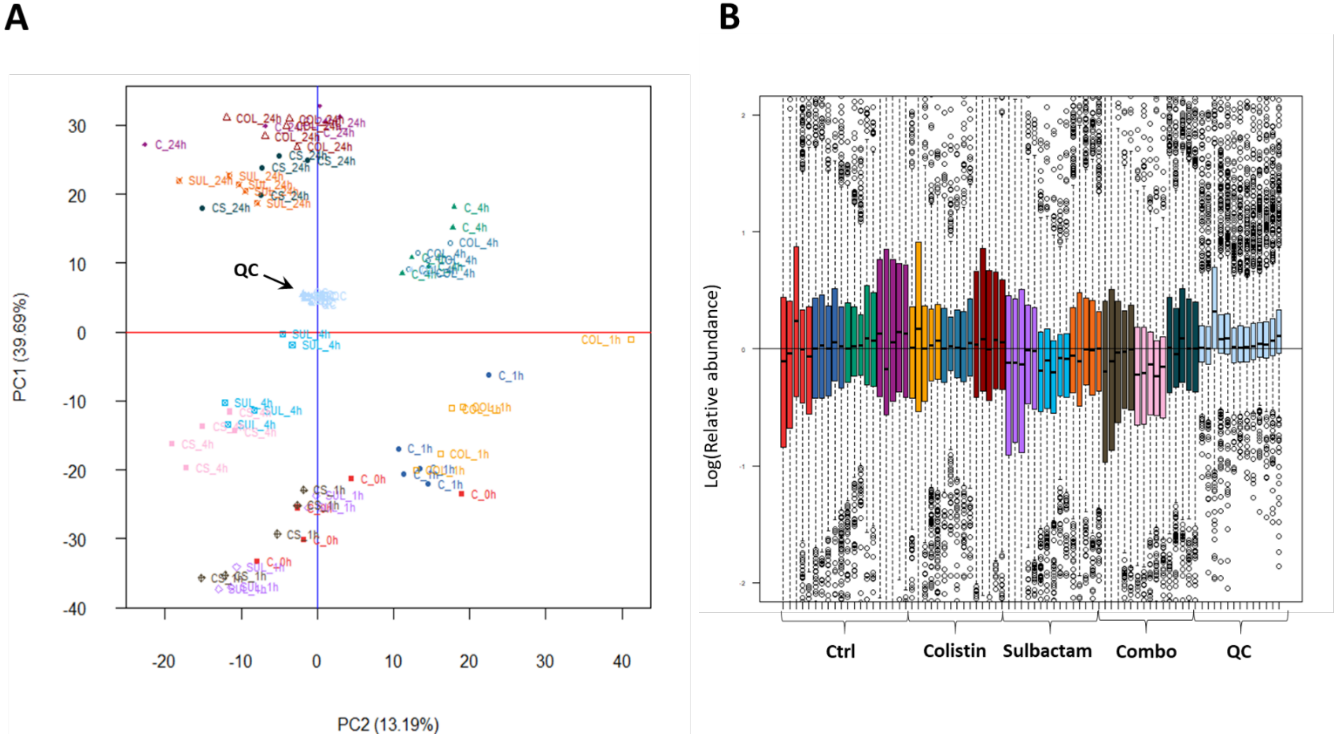

C

Treatment group	Median RSD (%)
Control (0 h)	27.2
Control (1 h)	20.1
Control (4 h)	15.9
Control (24 h)	21.9
Colistin (1 h)	25.6
Colistin (4 h)	13.8
Colistin (24 h)	16.6
Sulbactam (1 h)	19.6
Sulbactam (4 h)	18.3
Sulbactam (24 h)	16.0
Combo (1 h)	17.4
Combo (4 h)	15.0
Combo (24 h)	16.8
QC	14.2

Figure S2. (**A**) PCA score plot for intracellular metabolites of *A. baumannii* AB090342 and QC samples; (**B**) Log-transformed relative abundance; and (**C**) Data precision represented as the median relative standard deviation (RSD) for all metabolites based on all replicates of each group. Four quality controls (QCs) were analyzed within the same LC-MS batch with all the samples. Each dataset represents five biological replicates.

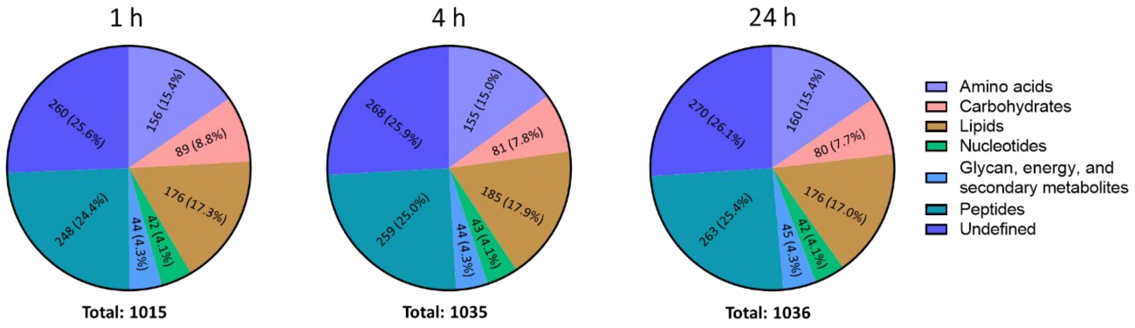

Figure S3. Total number of putative metabolites in each metabolic pathway in *A. baumannii* AB090342 at 1, 4, and 24 h. Metabolomics samples were analyzed using the HILIC method and all detected metabolites were putatively identified and quantified in IDEOM.

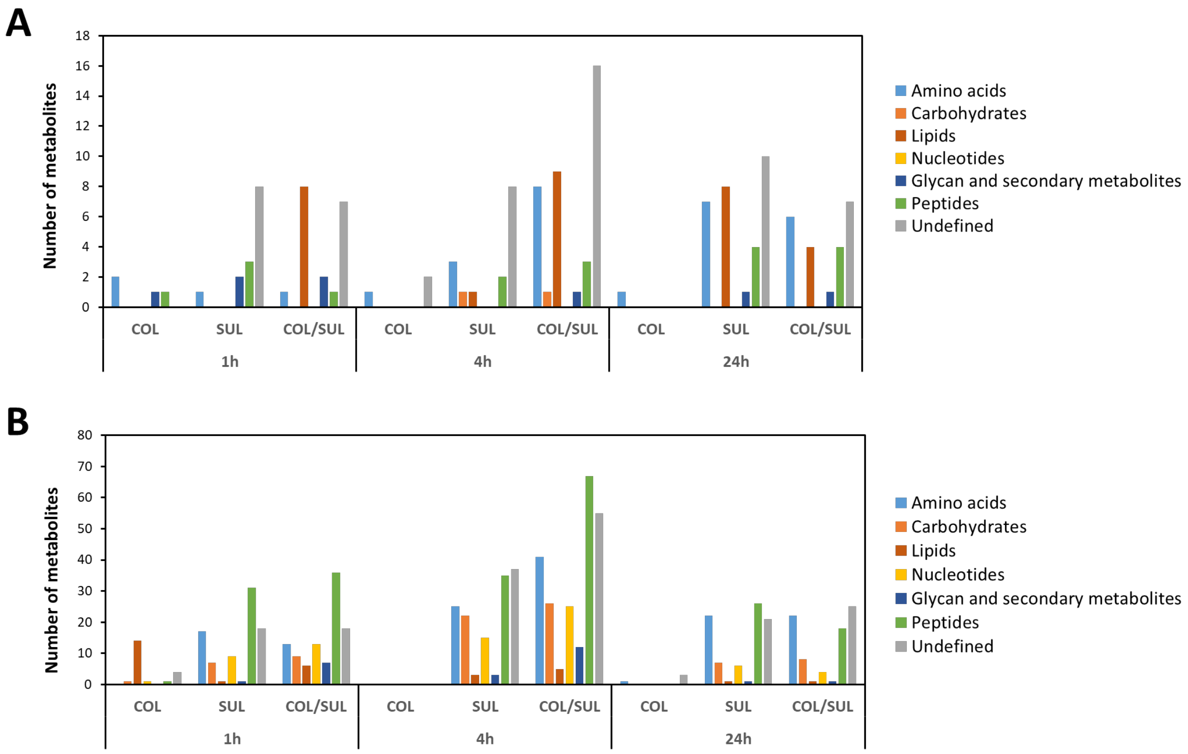

Figure S4. Numbers of significantly **(A)** increased and **(B)** decreased metabolites in major metabolic pathways in *A. baumannii* AB090342 due to treatments with colistin alone (COL), sulbactam alone (SUL) and the combination (COL/SUL) at 1, 4, and 24 h (FDR < 0.05 and $\log_2$ (fold change) ≥1 or ≤ -1 , one-way ANOVA).

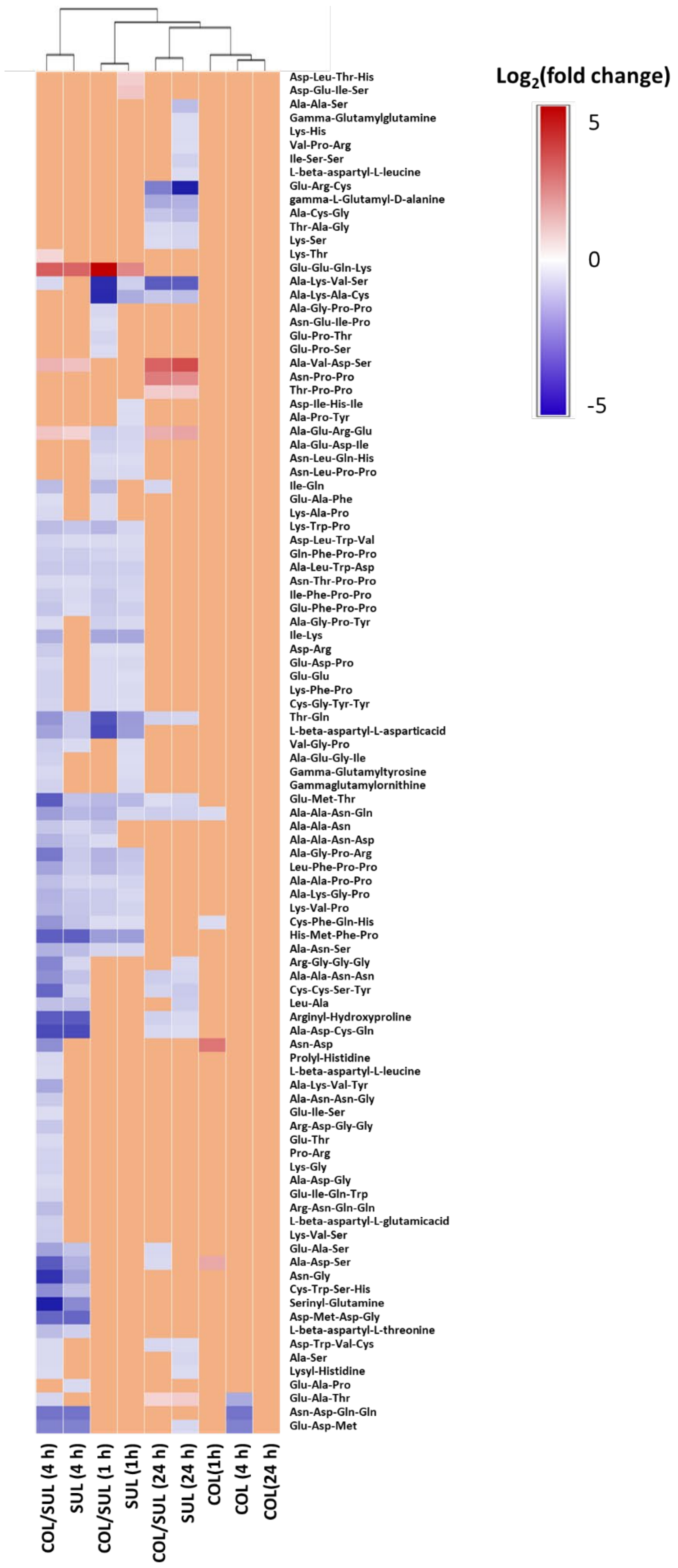
Log2(fold change)
5
0
-5
Asp-Leu-Thr-His
Asp-Glu-Ile-Ser
Ala-Ala-Ser
Gamma-Glutamylglutamine
Lys-His
Val-Pro-Arg
Ile-Ser-Ser
L-beta-aspartyl-L-leucine
Glu-Arg-Cys
gamma-L-Glutamyl-D-alanine
Ala-Cys-Gly
Thr-Ala-Gly
Lys-Ser
Lys-Thr
Glu-Glu-Gln-Lys
Ala-Lys-Val-Ser
Ala-Lys-Ala-Cys
Ala-Gly-Pro-Pro
Asn-Glu-Ile-Pro
Glu-Pro-Thr
Glu-Pro-Ser
Ala-Val-Asp-Ser
Asn-Pro-Pro
Thr-Pro-Pro
Asp-Ile-His-Ile
Ala-Pro-Tyr
Ala-Glu-Arg-Glu
Ala-Glu-Asp-Ile
Asn-Leu-Gln-His
Asn-Leu-Pro-Pro
Ile-Gln
Glu-Ala-Phe
Lys-Ala-Pro
Lys-Trp-Pro
Asp-Leu-Trp-Val
Gln-Phe-Pro-Pro
Ala-Leu-Trp-Asp
Asn-Thr-Pro-Pro
Ile-Phe-Pro-Pro
Glu-Phe-Pro-Pro
Ala-Gly-Pro-Tyr
Ile-Lys
Asp-Arg
Glu-Asp-Pro
Glu-Glu
Lys-Phe-Pro
Cys-Gly-Tyr-Tyr
Thr-Gln
L-beta-aspartyl-L-asparticacid
Val-Gly-Pro
Ala-Glu-Gly-Ile
Gamma-Glutamyltyrosine
Gammaglutamylornithine
Glu-Met-Thr
Ala-Ala-Asn-Gln
Ala-Ala-Asn
Ala-Ala-Asn-Asp
Ala-Gly-Pro-Arg
Leu-Phe-Pro-Pro
Ala-Ala-Pro-Pro
Ala-Lys-Gly-Pro
Lys-Val-Pro
Cys-Phe-Gln-His
His-Met-Phe-Pro
Ala-Asn-Ser
Arg-Gly-Gly-Gly
Ala-Ala-Asn-Asn
Cys-Cys-Ser-Tyr
Leu-Ala
Arginyl-Hydroxyproline
Ala-Asp-Cys-Gln
Asn-Asp
Prolyl-Histidine
L-beta-aspartyl-L-leucine
Ala-Lys-Val-Tyr
Ala-Asn-Asn-Gly
Glu-Ile-Ser
Arg-Asp-Gly-Gly
Glu-Thr
Pro-Arg
Lys-Gly
Ala-Asp-Gly
Glu-Ile-Gln-Trp
Arg-Asn-Gln-Gln
L-beta-aspartyl-L-glutamicacid
Lys-Val-Ser
Glu-Ala-Ser
Ala-Asp-Ser
Asn-Gly
Cys-Trp-Ser-His
Serinyl-Glutamine
Asp-Met-Asp-Gly
L-beta-aspartyl-L-threonine
Asp-Trp-Val-Cys
Ala-Ser
Lysyl-Histidine
Glu-Ala-Pro
Glu-Ala-Thr
Asn-Asp-Gln-Gln
Glu-Asp-Met
COL/SUL (4 h)
SUL (4 h)
COL/SUL (1 h)
SUL (1h)
COL/SUL (24 h)
SUL (24 h)
COL(1h)
COL (4 h)
COL(24 h)

Figure S5. Clustered heatmap profiles of the relative abundance for significantly affected peptides in *A. baumannii* AB090342 by all three treatments (colistin alone [COL], sulbactam alone [SUL] and the combination [COL/SUL]) at 1, 4, and 24 h, compared to the untreated control samples. Blue = significantly decreased ($\log_2$ FC $\leq$ -1), red = significantly increased ($\log_2$ FC $\geq$ 1), and orange = not significant.

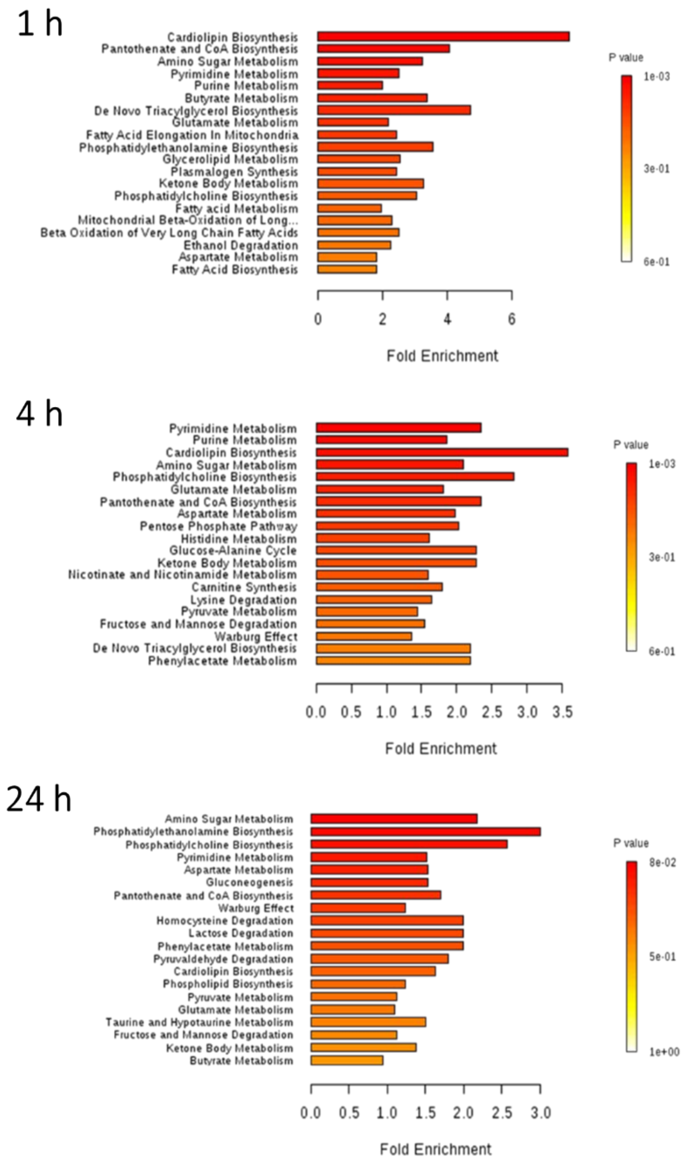

Figure S6. Pathway-associated metabolite enrichment analysis (top 20 most abundant metabolites) in *A. baumannii* AB090342 in response to colistin alone, sulbactam alone and the combination at **(A)** 1 h, **(B)** 4 h and **(C)** 24 h (FDR < 0.05, and $|\log_2 FC| \geq 1$, one-way ANOVA).

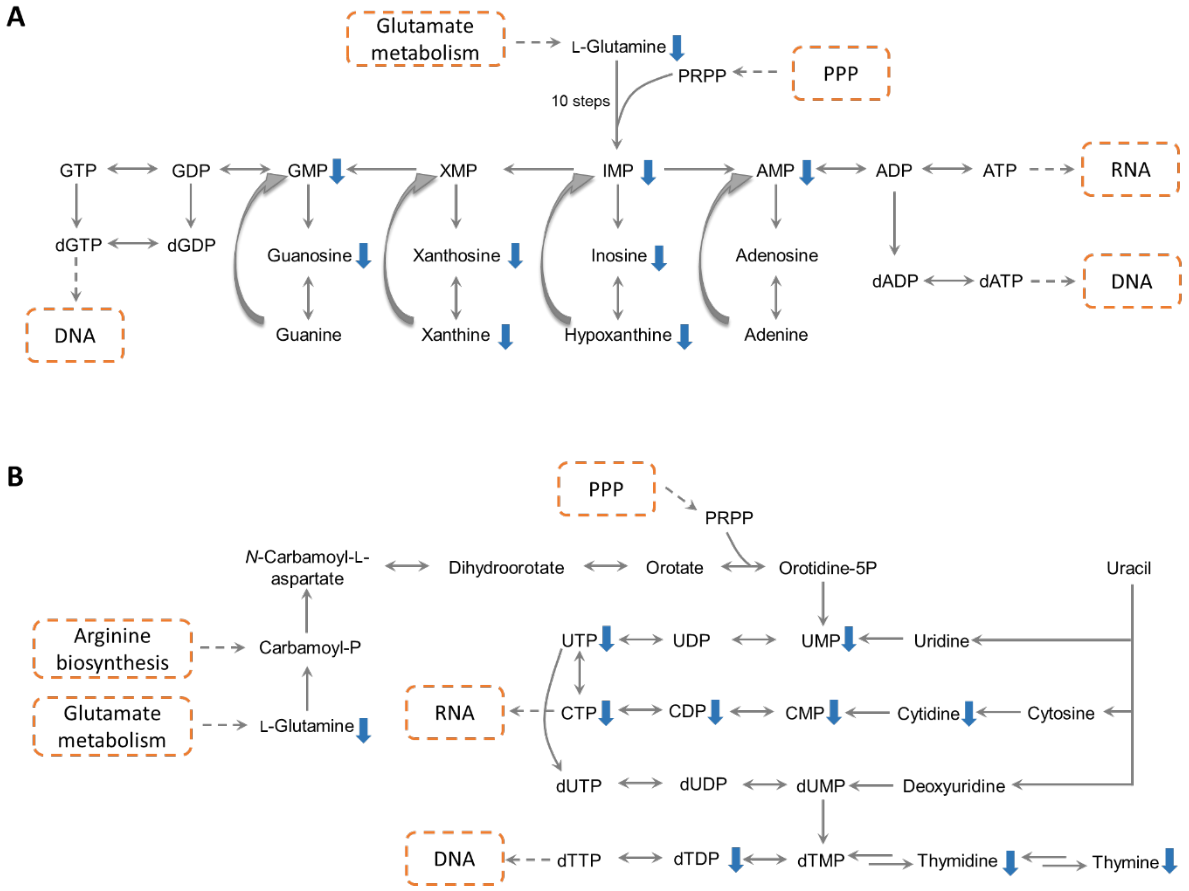

Figure S7. Sulbactam alone and the combination decreased most metabolite levels in **(A)** purine and **(B)** pyrimidine metabolism in *A. baumannii* AB090342 at 4 h. Metabolomics data were collected from five biological replicates per condition. Blue arrows indicate significantly decreased nucleotide metabolites (FDR < 0.05, $\log_2$ FC ≤ -1, one-way ANOVA).

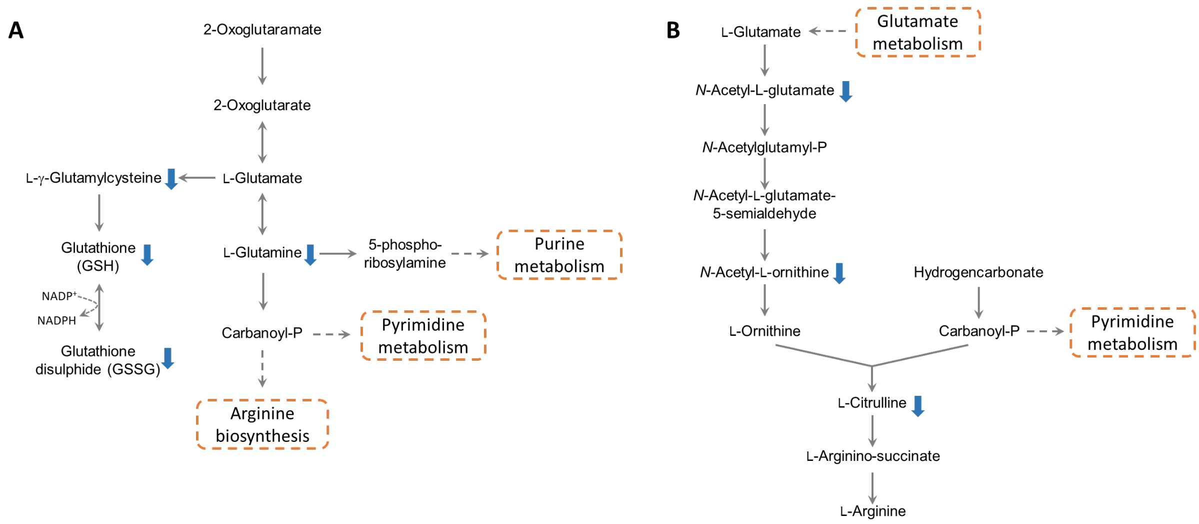

Figure S8. Sulbactam alone and the combination decreased metabolite levels in **(A)** glutamate metabolism and **(B)** arginine biosynthesis in *A. baumannii* AB090342 at 4 h. Blue arrows indicate significantly decreased metabolites. FDR < 0.05 and $\log_2$ FC ≤ -1 (one-way ANOVA).

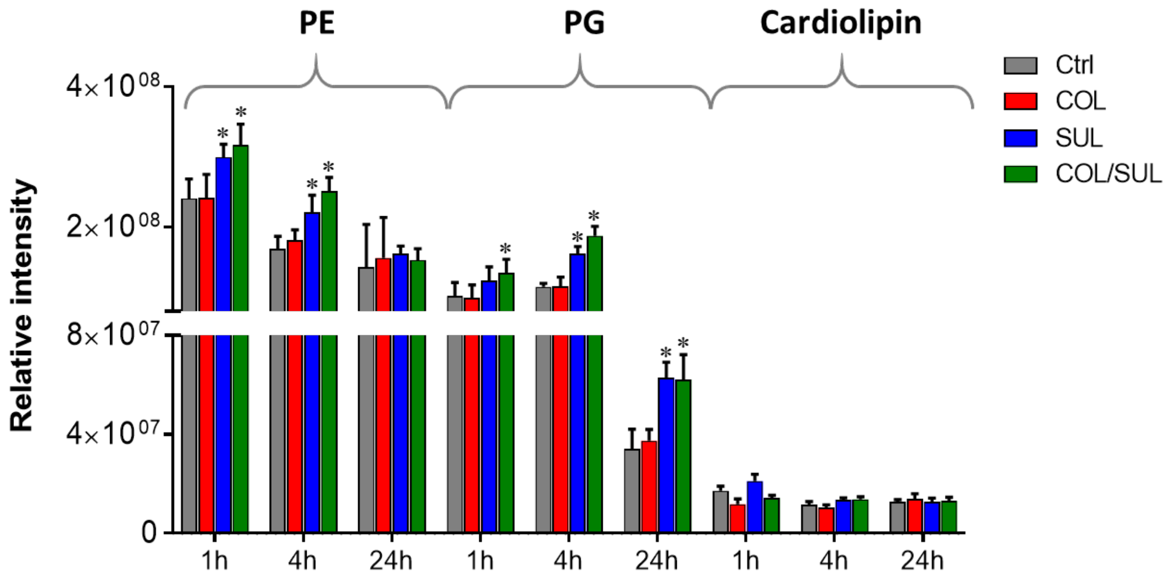

Figure S9. Relative intensities of total PE, PG and cardiolipin in *A. baumannii* AB090342 following treatments with colistin alone (COL), sulbactam alone (SUL) and the combination (COL/SUL) at 1, 4, and 24 h. Five biological replicates were employed and data were collected from HILIC chromatography and ESI-MS method. Asterisks represent statistical analysis between each treated group and the untreated control (*$p < 0.05$, student's t test).
